# Solidified Formaldehyde

**Published:** 1902-10-15

**Authors:** F. B. Lawrence

**Affiliations:** Eldorado, Ka.


### SOLIDIFIED FORMALDEHYDE.

BY F. B. LAWRENCE, D.D.S., ELDORADO, KA.

Formic aldehyde is a gas produced by the oxidation of wood alcohol, which by virtue of its specific gravity being the same as atmospheric air, searches out and destroys all forms of bacteria antagonistic to health, thoroughly tanning the protoplasm of the cell and sealing forever from proliferation the enclosed nucleus. This gas is now almost exclusively used for disinfection after epidemics and to suppress and mitigate existing cases. Seeking, as a ferret seeks his prey, in each crack and cranny for the death-bringing germs and destroying the same if found in the presence of moisture.

Apparatus has been given to dentists with which to apply the gas to the root-canal direct, but had to be discarded, as the application could not be sufficiently prolonged to produce thorough asepsis, and if inhaled would strangle the patient. Its aqueous solution was then tried. In this form it is almost a caustic, killing all tissue it comes in contact with (apparently after the manner of arsenious acid), followed by sloughing and exfoliation. This formalin would if it came in contact with the gums, or was forced through the apex of the root, cause great pain, and when this took place, you might consider yourself lucky if you lost but the one tooth. This drug has been virtually discarded through want of method of application devoid of danger to healthy tissues.

In 1897 I went to my druggist for wood alcohol, with which to produce formaldehyde to disinfect my office. He gave me instead a jar of polymerized formaldehyde. This is produced by heating the aqueous solution, when the gas, instead of evaporating, becomes polymerized as soon as the concentration exceeds 40 percent it increases with the density of the liquid, until becomes solid and then is very prone to ignite. In this form it almost loses its caustic action and may even be administered in as high as 30-grain doses. This polymerized formaldehyde was in my medicine cabinet when a case presented—a superior right canine which had baffled all my attempts to bring it to a condition for filling. The pulp had been dead for months, and presented every indication of a blind abscess. I concluded that the formaldehyde in this form was indicated. I placed about one-half a grain within the pulp-chamber and sealed with cement to keep same from coming in contact with the gums, and to force the gas through the apex of the root. Contrary to the patient's custom of returning next morning after the canal was closed, with pain located at apex of the root, she did not return for several days. That root is now acting as an abutment for a bridge. From that moment I discarded all other methods of treating devitalized teeth and pinned my faith to formaldehyde, and not a case have I lost that I know of where the patient followed my directions.

One fact you may lay down; you must not place formaldehyde in its aqueous solution or in this formation against any live tissue, unless you desire an excruciating pain for several hours. If it is placed within the tooth, the pain will subside only after it has become loosened and very sore to the touch, and the tooth will be reinstated to its normal condition and again perform the functions which the creator intended; but in the meantime, you will have to stand your patient off with all the smiles and good words you have. So don't put it in direct application to any live tissue, even though it may be a little fiber of nerve left the canal that you find it hard to reach and devitalize; if you don't devitalize it you will have trouble, and if you do you will have no trouble. Another fact that you must bear in mind, have the canal absolutely free from all debris, as far as instrumentation will perform the same. Don't have the canal filled with water or pus; if you do, this same water or pus will dissolve the gas from the polymerized formaldehyde until it becomes an irritant solution. Have the canal opened and moist not dry, the formaldehyde gas will not be generated where everything is dry, it must have moisture.

In devitalizing a pulp take a little piece of asbestos paper, and on one side of that place a coating of nerve paste. I have had the best results by mixing that with tannic acid dissolving in a minum of sanitol. Moisten the asbestos with carbolic acid, first just enough to moisten it, and touch the opposite side with formaldehyde. Place that over your pulp, flow your cement in that it may perfectly seal the same, and you will have a devitalized pulp with little or no pain. You will also have a condition, where if circumstances arise that the patient can not return for days or weeks, or possibly for two or three months, he will have no bad effects whatever or any pain.

The formaldehyde or formic aldehyde in the tooth decomposes so slowly that it causes no irritation whatsoever to the sensitive portion of that tooth, but is enough to keep the contents from becoming septic.

A gentleman called on me and said he was having trouble. I found a right inferior central with a dead pulp, and an abscess just forming; a little swelling at the apex of the tooth very sore to the touch. The tooth itself was sore; I could hardly touch it with the instrument; but I did succeed in opening it, and with thorough instrumentation, I cleaned the canal with a fine broach, the apex was open and a drop or two of pus exuded. I cleaned that out with paper points, getting all I could. The pain increased instead of diminishing. With the aqueous solution you would hesitate to do this, I took a small piece of this formaldehyde, which is Leininger's, about twice the size of the head of a pin—probably a half-grain, hardly that, and dropped that in the canal, carefully pressing it with the instrument, just to place it and flowed cement over it. That I believe was six or eight months ago. The application of the formaldehyde in this case suppressed the pain in less than three hours. By evening he was free and has never returned for treatment.

This Leininger's formaldehyde, turning to gas, will work its way through the apex, and if you have pus or pathogenetic germs beyond the apex, will search them out, it will also follow the tubuli to the cementum. It will thoroughly embalm the canal contents.

Another case that I had but a few months ago, and some of these cases happened when I first began the use of formaldehyde was the left inferior first molar. It was so loose, there seemed to be no attachment whatever. The tooth was aching a very little. There were five or six fistulous opening on the gum on both the buccal and lingual surfaces. The gum itself was a dark color, threatening what appeared, to me at least, to be gangrene and possibly necrosis of the process. I opened the canal and remove the debris, which was principally pus and places this formaldehyde in position, sealed the same, and I give you my word in less than two days I could not find an opening on the gums. That was about six months ago, and the gentleman today is grinding on that molar as though it had never been in anything but a normal condition.

These two cases I am giving you among dozens, that I have treated in the same way during the last two or three years; and in that time I do not say I have had no trouble, because I have. I remember one case, I applied it in a molar for a young lady. She returned about two days afterwards, the tooth not hurting, but her gum looked exactly to me as though there were in the neighborhood of a hundred fistulous openings. I thought that I had not only lost that tooth, but the chances were I was going to have trouble with the maxilla. I attempted to enter some of the fistulous openings, and could not, they were not open. It was simply an outward manifestation of an inward condition that did not exist. All I could think of was to apply iodine and aconite. In about a week after that lady returned ; I filled the root and the tooth is still there. This happened nearly three years ago and she is still grinding upon the same devitalized tooth.

Another case that I wish to present to you that is of practical interest. About three years ago a young lady came to me with the superior left lateral crown gone. The apex of the root had absorbed until I could pass a hoe excavator through the canal and turn it around and catch it on the end of the root. The moment I touched it, it bled. She was very desirous of having a crown attached to that root. The root itself was out of the arch, but the crown could be placed so as to make the arch symmetrical. I succeeded in saving that root, and it is still doing good service. I put the formaldehyde in there and knew I must not get it through the apex of the root. I closed it and a day or two after when she returned, I cleaned it out and the hemorrhage began again. I closed it again with a piece of cotton with a little vaseline and formaldehyde on it and forced it to the end of the root and a day or two afterwards opened it. It bled again and again I packed the cotton in there with the vaseline and formaldehyde mixed. In about a week after that time, when she returned by appointment, I put the root-filling that I use into the root with a large gutta-percha point, clipping the edge of it and trying it until it would just come in contact with the sensitive structures. I left it three weeks or so, and when she returned I crowned that root and it is still there.

Another case was a man who has never been able to retain a dead tooth ; he has had three or four treated and every one of them had to be extracted. This was also a right inferior lateral. I treated with the formaldehyde, closed it up as I had done before. He returned in a day or two afterwards complaining of pain ; I opened it, and it bled, pus and effluvia came from the apex of the root. I treated it again with thorough cleansing and closed it again. I did not give the desired result ; then I treated same with a dressing of glyco-thymolin and filled it. That was two or three yeas ago and he still has that tooth with the crown attached.

One place you will find this polymerized formaldehyde is very difficult to use indeed—in a tooth where there is a dry gangrene. I treated one unsuccessfully until the thought came to me that this formaldehyde would not work in a dry place, and only in the presence of moisture ; I moistened that root-canal thoroughly, then removed only the excess with a paper point, that I might not have a 40 per cent aqueous solution in the tooth, then placed my formaldehyde and gave it one treatment with thorough instrumentation, and a few days afterwards filled the root and crowned it and it is still there.

I take issue with the accustomed methods of filling root-canals, which is essentially a part of the successful use of this method of treating pulp canals. You haven't a method of filling a root-canal now that thoroughly and positively seals the apex of the root from all seeping. I make that statement, not from my own tests, but from a report made, I believe it was, by the Odontological Society in Chicago.

With every known method of filling a root-canal, with chlora-percha, with gutta-percha, with phosphates, with gold, with copper points—every method you have, you can take the tooth and put it in a solution of aniline ink, and your chlora-percha or gutta-percha point will leak half-way down your canal in less than forty-eight hours. Simply because the cloroform evaporates from the chlora-percha and in evaporating there is shrinkage and you can not get perfect adaption to the walls of your canal to save your neck. It is impossible.

With this method, that I have used for filling nerve-canals, you can fill a nerve-canal, drop the tooth in the aniline ink, and leave it there months and you will have no seeping from the apex.

Now, allow me to state the method I have used for filling these nerve-canals. First, don't dry your tooth too much, leave the natural moisture there, to make sure the formaldehyde has thoroughly embalmed or put out of use any germ that does not belong there, that it is dead beyond resurrection.

Use vaseline to make a paste of the periform thoroughly mix and incorporate the periform with that vaseline. It does not dissolve, but does become like a mass, thoroughly permeating the vaseline. Fill your root-canal with this, being careful not to force it beyond the apex of the root, and seal it in place. It will produce little or no irritation on account of the vaseline so thoroughly surrounding and incapsuling every atom of the periform. After you have filled the root, take a gutta-percha point and push one of the finest up in the canal until you are sure you are at the apex of the root, possibly the sense of touch will enable your patient to tell you that. If it does not fill the root-canal, take another small one and follow it up in size, one after another. The excess of this vaseline mixed with your periform will exude into the cavity; then with a very hot burnisher, after wiping the excess out with cotton, seal the same; and if you desire to put a crown on that tooth, don't hesitate. Use a bur larger than your nerve-canal with a pretty good motion to produce sufficient heat from friction to soften the gutta-percha, and really it only softens it at the point of contact with your bur. Revolve that rapidly, and open your canal. The moment the patient flinches, or shows there is any pressure, cease; using one instrument or another until you can have that sufficiently open to a depth sufficient to retain the post that you may have on that crown.

Use it in your cuspidor. Some men that I know cleanse and wash their cuspidors every ten minutes during the day. Others have fountain spittoons, which I am told, by one man at least, will become very offensive. The drain-pipe will become offensive and you can not tolerate it in the office. Pour a solution of formaldehyde in there and let it go through and you will never have a stench from your cuspidor or fountain spittoon.

While the liquid formalin can not be applied to sensitive dentine, where the indications are that the pulp should not be devitalized, and you desire to keep the same, place a nerve-capping of any of the cements, save archite and petroid, by first mixing with the liquid of the cement approximately one-tenth of periform, thoroughly rubbing same together before mixing the powder with the liquid; flow over the bottom of the cavity, let harden, trim excess, prepare cavity, as you may and fill with the material desired, with the assurance that aside from possibly a few throbbing pains of no import, the tooth will retain its vitality indefinitely.

Every dental office, as every drug store has an effluvia peculiar to itself. Sterilize your office thoroughly once a week. Saturday night is a good time for this. Close the office windows and the door and burn at least an ounce of "glutol" in one of the generators made for it, or burn your lamp if you see fit; but in the oxydation of the wood alcohol you are not certain what you are getting, you may get what you are after and you may not. Using glutol you will find your office Monday morning, when you open it, like a May day after a shower, absolutely sterilized and cleansed.

The use of the generator in the office eliminates every evidence to the olfactory nerves of drugs, and so forth, and leaves the room with the same sense of freshness and cleanliness, which pervades the atmosphere of a June morning after a refreshing rain, and you have the absolute assurance that all germs of any contagious disease which may have been brought to the office by careless patients are positively destroyed, and carrying same to your home or to the homes of others, is absolutely impossible.

In closing, I am not recommending this formaldehyde to the tyro or the quack, but to the legitimate practitioner who seeks for quicker and better methods of reaching the desired end. Judgment from my knowledge of the effect of this wonderful embalmer, germicide and deodorizer compels me to believe that in solidified formaldehyde is found the *sine qua non* for successful treatment of all conditions of devitalized and abscessed teeth and their sequelæ.--Abstract from August *Western Journal*.

